# Expression and Activity of Fyn Mediate Proliferation and Blastic Features of Chronic Myelogenous Leukemia

**DOI:** 10.1371/journal.pone.0051611

**Published:** 2012-12-17

**Authors:** Melissa M. Singh, Adrienne Howard, Mary E. Irwin, Yin Gao, Xiaolin Lu, Asha Multani, Joya Chandra

**Affiliations:** 1 Department of Pediatrics Research, The University of Texas M.D. Anderson Cancer Center, Houston, Texas, United States of America; 2 Molecular Cytogenetics Core Facility, The University of Texas M.D. Anderson Cancer Center, Houston, Texas, United States of America; 3 Graduate School of Biomedical Sciences, The University of Texas at Houston Health Science Center, Houston, Texas, United States of America; Mayo Clinic College of Medicine, United States of America

## Abstract

The BCR-ABL1 oncogene is a tyrosine kinase that activates many signaling pathways, resulting in the induction of chronic myeloid leukemia (CML). Kinase inhibitors, such as imatinib, have been developed for the treatment of CML; however, the terminal, blast crisis phase of the disease remains a clinical challenge. Blast crisis CML is difficult to treat due to resistance to tyrosine kinase inhibitors, increased genomic instability and acquired secondary mutations. Our recent studies uncovered a role for Fyn in promoting BCR-ABL1 mediated cell growth and sensitivity to imatinib. Here we demonstrate that Fyn contributes to BCR-ABL1 induced genomic instability, a feature of blast crisis CML. Bone marrow cells and mouse embryonic fibroblasts derived from Fyn knockout mice transduced with BCR-ABL1 display slowed growth and clonogenic potential as compared to Fyn wild-type BCR-ABL1 expressing counterparts. K562 cells overexpressing constitutively active Fyn kinase were larger in size and displayed an accumulation of genomic abnormalities such as chromosomal aberrations and polyploidy. Importantly, loss of Fyn protected mouse embryonic fibroblast cells from increased number of chromosomal aberrations and fragments induced by BCR-ABL1. Together, these results reveal a novel role for Fyn in regulating events required for genomic maintenance and suggest that Fyn kinase activity plays a role in the progression of CML to blast crisis.

## Introduction

Chronic myeloid leukemia (CML) is characterized by an acquired genetic abnormality, a t(9;22) (q34;q11) reciprocal translocation, that results in the expression of the BCR-ABL1 oncogene [1], [2], [3]. The disease develops in three phases; chronic, accelerated, and blast crisis. Progression of CML from chronic phase to blast crisis is associated with enhanced cell proliferation and survival, blockage of differentiation, and diminished apoptosis [4], [5]. Additionally, blast crisis CML cells display increased genomic instability [6], [7] and accumulation of secondary mutations [5], [8]. The combination of these cellular features results in the terminal phase of disease that is refractory to current therapies, including small molecule kinase inhibitors such as imatinib. Further understanding of the molecular events altered by unregulated BCR-ABL1 signaling will aid in the design of novel therapeutics aimed at targeting refractory and blast crisis CML.

BCR-ABL1 activates a variety of signaling pathways that contribute to malignant transformation in CML, including the Src family of kinases. Reports demonstrate increased activation of several Src family members such as Src, Lyn, and Hck by BCR-ABL1 [9], [10]. Moreover, gene knockout studies support a role for these proteins in BCR-ABL1 mediated disease [11], [12], [13], [14]. However, BCR-ABL1 expression in bone marrow cells derived from triple knockout mice deficient in Hck, Lyn, and Fgr still effectively induced CML [11]. These results suggest the potential involvement of other Src kinase family members in CML disease progression and resistance.

Fyn is a Src family member that is ubiquitously expressed and known to function in T-cell signaling and differentiation [15], [16], entry into mitosis [17], and cell adhesion [18]. While several studies have evaluated the role of Src family members in CML, few have specifically studied the role of Fyn. Microarray analysis of phase-specific CML revealed that upregulation of the Src kinase family member Fyn was linked to imatinib resistance [19]. Moreover, Fyn was identified as a hub for signaling in BCR-ABL1 expressing acute lymphoblastic leukemia (ALL) specimens, a disease that resembles blast crisis CML [20]. Our published results demonstrate elevated Fyn protein and mRNA in BCR-ABL1 expressing cells [21]. We also found that Fyn expression was higher specifically in blast crisis CML patient specimens when we compared each phase of disease with specimens from non-CML patients [21]. Upregulation of Fyn was also evident in a mouse model for CML and knockdown of Fyn slowed the growth of K562 cells in an in vivo xenograft model of leukemia [21]. Fyn transduces mitogenic signals in CML and knockdown of Fyn in a human blast crisis CML line, K562, display slowed growth kinetics and increased sensitivity to imatinib [22]. In addition, in gene expression profiling studies in imatinib resistant CML cells, Fyn was identified as an important gene in conferring resistance to BCR-ABL1 inhibitors [23]. Taken together, these data implicate Fyn as a mediator of CML progression and sensitivity to kinase inhibitors.

**Figure 1:**
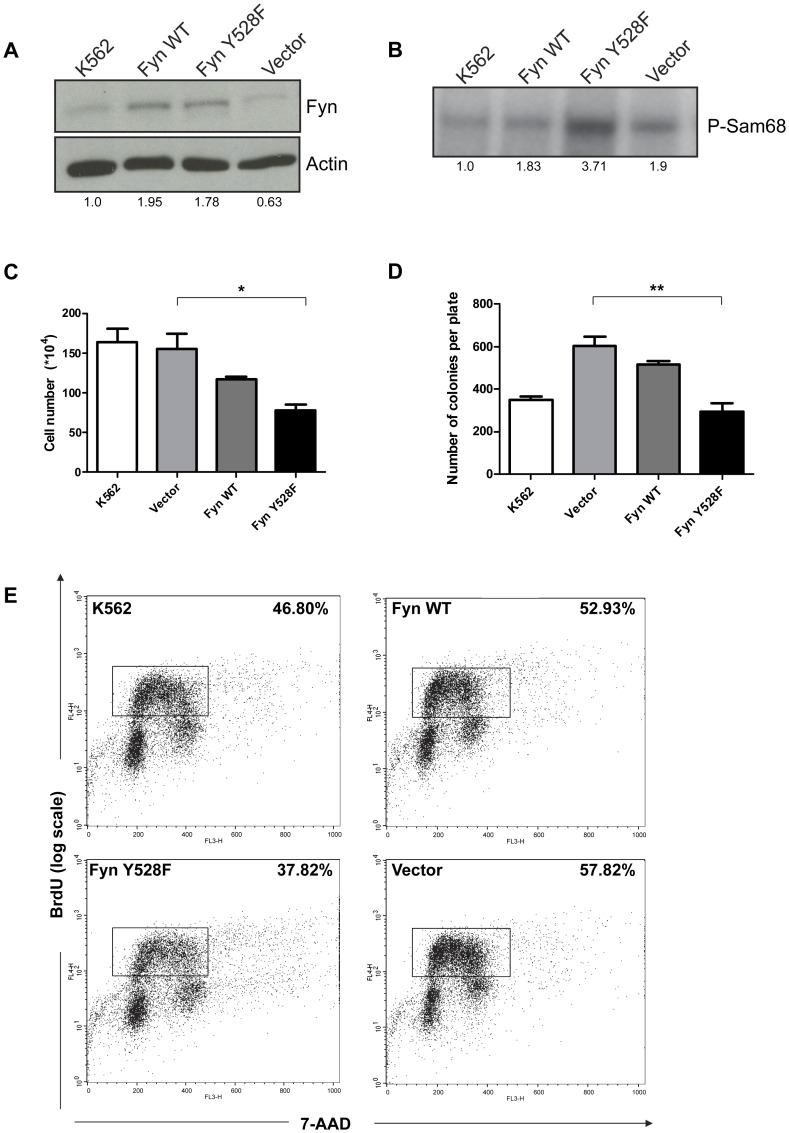
Overexpression of constitutively active Fyn kinase in K562 cells decreases cell growth. K562 cells were transduced with vector, wild-type (Fyn WT), and constitutively active Fyn (Fyn Y528F). (A) Fyn protein expression was evaluated in total cell lysates (100 µg) by Western blot analysis using an antibody specific for Fyn. Actin was used as a loading control. Densitometry was performed using ImageJ and the blot is representative of three independent experiments. (B) Protein lysates from K562 cells transduced with vector, wild-type Fyn, and constitutively active Fyn were subjected to immunocomplex kinase assays using Sam68 as a substrate. Phosphorylated Sam68 was detected by autoradiography. The results are representative of three independent experiments. (C) Fyn overexpressing K562 cells were plated at 100,000 cells/mL in a 12 well plate and viable cells were counted after 72 hours. *p<0.01, n = 3, mean+SEM. (D) Fyn overexpressing K562 cells lines were suspended in MethoCult GF H4434 medium and colonies were counted five days after plating. **p<0.001, n = 3, mean+SEM. (E) Fyn overexpressing K562 cells were pulsed for 30 minutes with 10 µM BrdU and double stained with an APC-conjugated BrdU antibody and 7-AAD for cell cycle. Dot plots are representative of three independent experiments.

In this manuscript, we evaluate the effects of Fyn kinase activity on the growth and survival of BCR-ABL1 expressing cells. Using Fyn knockout mice, we demonstrate a requirement for Fyn in the growth and clonogenicity of BCR-ABL1 expressing cells. In addition, we show that constitutive activation of Fyn leads to increased cell size and genomic abnormalities, a hallmark of blast crisis CML. Additionally, BCR-ABL1 induced genomic instability is reduced in Fyn-deficient mouse embryonic fibroblast cells. Collectively, our data link Fyn expression and activity to increased growth, proliferation, and genomic instability, all of which are features of blast crisis CML which remains a clinical challenge due to limited effective treatment options.

## Materials and Methods

### Ethics Statement

All animal experiments were approved by the UTMDACC Institutional Animal Care and Use Committee (03–04–02933) and were performed in accordance with the guidelines for the Care and Use of Laboratory Animals published by the National Institutes of Health.

**Figure 2:**
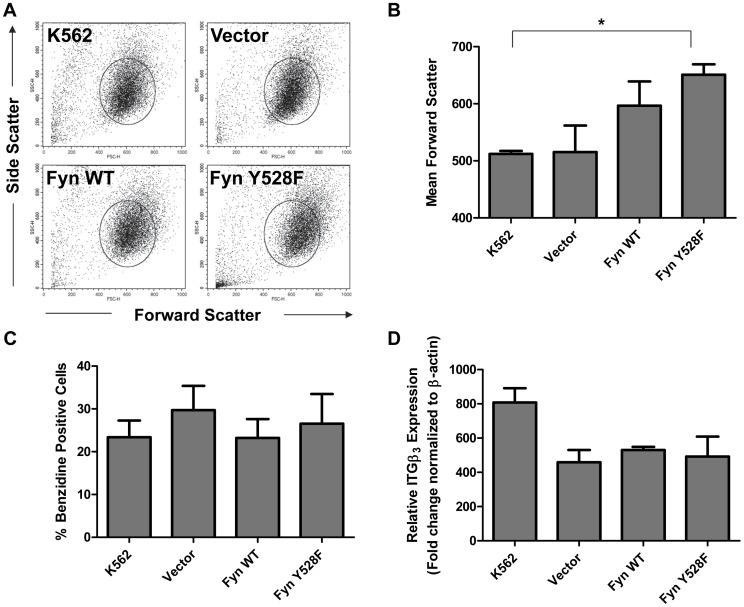
Increased size of constitutively active Fyn overexpressing cells is not a result of differences in differentiation. (A) Forward and side scatter dot plots were used to determine changes in cell size and granularity, respectively, among the K562 cells transduced with vector, wild-type (Fyn WT), and constitutively active Fyn (Fyn Y528F). The circle represents the live cell population for K562 cells. Data are representative of three independent experiments. (B) Graphical representation of the mean forward scatter values obtained from the live cell population from the dot plots in A. *p<0.05, n = 3, mean+SEM. (C) Fyn expressing K562 cells were treated with 40 µM hemin for 72 hours. Erythroid differentiation was determined by benzidine staining and the number of positive cells was counted by light microscopy. A minimum of 200 cells per condition were counted. n = 3, mean+SEM. (D) Fyn expressing K562 cells were treated with 10 nM PMA for 72 hours and RT-qPCR was performed to evaluate the level of integrin β_3_ (ITGβ_3_) expression. Ct values are normalized to β-actin and fold change is relative to the expression of ITGβ_3_ in untreated K562 cells. Each sample was run in duplicate. n = 2, mean+SEM.

### Chemicals and Cell Lines

All chemicals, unless otherwise noted, were purchased from Sigma Aldrich (St. Louis, MO). Fyn wild-type and Fyn knockout mice were purchased from Jackson Laboratories (Bar Harbor, ME). Bone marrow cells were harvested as previously described [21] and maintained in Iscove’s modified Dulbecco medium supplemented with 15% fetal bovine serum (FBS), 0.1% bovine serum albumin (BSA), 25 µM β-mercaptoethanol, 100 U/ml penicillin/streptomycin, 100 IU/mL recombinant murine IL-3, 100 IU/mL recombinant murine IL-6, and 1000 IU/mL (100 ng/mL) recombinant murine stem cell factor (SCF) (all obtained from PeproTech, Rocky Hill, NJ) [24]. Murine growth factor-dependent pro-B lymphoid BaF3 cells transformed with empty vector (BaF3 vector) or transformed with wild type p210 BCR-ABL1 (BaF3 p210) were kindly provided by Dr. Charles Sawyers (Memorial Sloan Kettering Cancer Center, NY) [25]. The K562 human chronic myelogenous leukemia cell line expressing BCR-ABL1 was obtained from American Type Culture Collection (Manassas, VA). K562 and BaF3 cells were grown in RPMI 1640 medium supplemented with 10% FBS, 2 mM L-glutamine, and 100 U/ml penicillin/streptomycin. BaF3 vector cells were supplemented with 1 ng/ml recombinant murine IL-3 (PeproTech, Rocky Hill, NJ). Immortalized mouse embryonic fibroblasts (MEFs) derived from Fyn wild-type and knockout mice were kindly provided by Paul Stein (Northwestern University, Chicago, IL) and maintained in DMEM supplemented with 10% FBS, 2 mM L-glutamine, and 100 U/ml penicillin/streptomycin. All cells were maintained at 37°C in a humidified environment containing 5% CO_2_.

**Figure 3:**
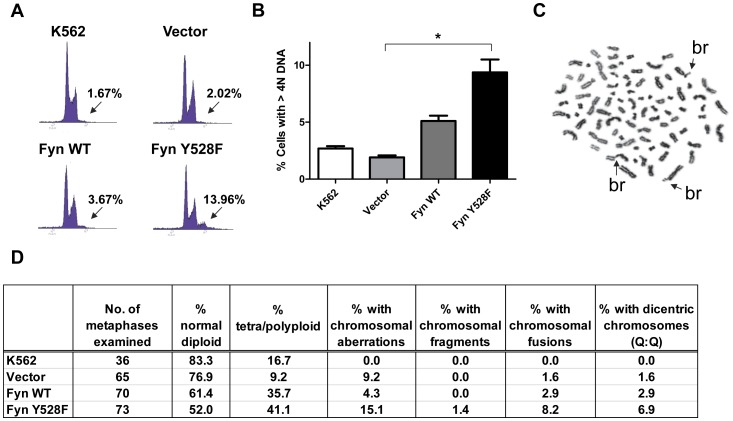
Increased Fyn kinase activity leads to the accumulation of genomic instability in CML cells. (A) Cell cycle profiles of K562 cells transduced with vector, wild-type (Fyn WT), or constitutively active Fyn (Fyn Y528F) were evaluated by propidium iodide staining and analysis by flow cytometry. Histograms are representative of three independent experiments. (B) Graphical representation of the percent of cells with greater than 4N DNA content. *p<0.01, n = 3, mean+SEM. (C) A representative metaphase spread from constitutively active Fyn expressing K562 cells. Chromatid breaks (br) are indicated by arrows. (D) Table depicting the various chromosomal abnormalities seen in Fyn overexpressing K562 cells.

### Construct Design and Retroviral Transduction

Fyn sequences were PCR amplified from K562 cells and cloned into the pRevTRE retroviral vector (CloneTech, Mountain View, CA). Constitutively active Fyn constructs were generated by creating a point mutation (Y528F) using one-time PCR with high fidelity DNA Polymerase. K562 cells were transduced with virus containing empty vector (Vector), wild type Fyn (Fyn WT), and constitutively active Fyn (Fyn Y528F). Following infection, GFP+ cells were sorted and maintained in culture containing 400 µg/ml G418.

**Figure 4:**
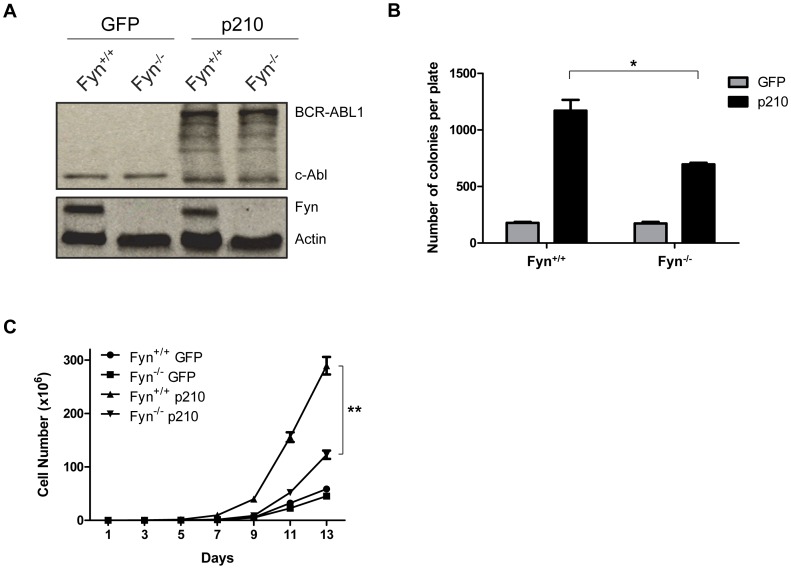
Fyn contributes to the growth and clonogenicity of BCR-ABL1 expressing bone marrow cells. (A) Bone marrow cells from wild-type (Fyn^+/+^) or Fyn knockout (Fyn^−/−^) mice were transduced with vector (GFP) or vector expressing p210 BCR-ABL1 (p210). Expression of p210 and Fyn was detected in total protein lysates (100 µg) using antibodies specific for Abl and Fyn, respectively. Actin was used as a loading control. The blot is representative of three independent experiments. (B) Transduced bone marrow cells were plated in methylcellulose soft agar and colonies were counted after five days. *p<0.01, n = 3, mean+SEM. (C) Transduced bone marrow cells were plated in 96-well plates at 150,000 cells/mL. Every two days, cells were stained with trypan blue and viable cells were counted to determine growth rate. *p<0.001, n = 5, mean+SEM.

### Growth Rate and Colony-Forming Assay

K562 cell lines were seeded at 500,000 cells per ml in a 12-well plate. After 24, 48, 72, and 96 hours, live cells were counted by trypan blue exclusion using a hemacytometer. Immortalized MEFs were plated at 50,000 cells per well of a 6-well plate and counted daily until the cells reached confluence. To determine clonogenic growth, a methylcellulose colony-forming assay was performed according to the manufacturer’s protocol (StemCell Technologies, Vancouver, BC). Each cell line, transduced K562 or bone marrow cells derived from Fyn knockout animals, was resuspended in Iscove’s modified Dulbecco’s medium with 2% FBS. One thousand cells in suspension were mixed with 1 ml MethoCult GF H4434. A syringe attached to a 16 gauge needle was used to dispense the MethoCult mixture into a 35 mm gridded tissue culture dish. Dishes were placed into larger 100 mm dishes with water to maintain humidity. After five days, the number of colonies was counted using a Leica light microscope.

**Figure 5:**
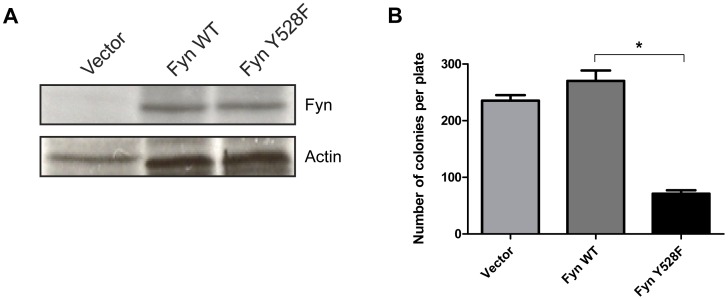
Overexpression of active Fyn decreases clonogenicity in the absence of BCR-ABL1. (A) Bone marrow cells from Fyn knockout mice were transduced with vector, wild-type Fyn (Fyn WT), or constitutively active Fyn (Fyn Y528F). Expression of wild-type and constitutively active Fyn was detected in total protein lysates (100 µg) using a Fyn specific antibody. Actin was used as a loading control. The blot is representative of three independent experiments. (B) Fyn reconstituted bone marrow cells were suspended in MethoCult GF H4434 medium and colonies were counted five days after plating. *p<0.001, n = 3, mean+SEM.

### Immunoprecipitation and Western Blot

Immunoprecipitation and Western blot were performed as previously described [21]. Cells were lysed overnight in buffer containing 50 mM Tris pH 7.4, 100 mM NaCl, 1 mM EDTA, 1% NP-40, 0.1% Triton X-100, and 1% β-mercaptoethanol at 4°C. Samples were centrifuged at 12,000 rpm, 4°C for 15 minutes and supernatant was collected. Protein concentrations of samples were determined using the BioRad DC protein assay (Molecular Devices, Sunnyvale, CA). For immunoprecipitation of Fyn, 500 µg protein lysate was incubated overnight at 4°C with 5 µl monoclonal Fyn antibody. Fyn complexes were pulled down by incubating samples at 4°C for 1 hour with 10 µl protein A beads. Loading buffer was added and samples were boiled for five minutes followed by loading of supernatant onto a 10% SDS-polyacrylamide gel and subsequent electrophoresis. The separated proteins were transferred to a Hyperbound-ECL nitrocellulose membrane. Membranes were blocked for 2 hours at room temperature with 5% milk in Tris-buffered saline-0.1% Tween (TBS-T) followed by overnight incubation with primary antibody (Fyn 1∶750, Src pY416 1:1000 (EMD Millipore, Billerica, MA) or actin 1∶1000 (Sigma, St. Louis, MO)) at 4°C. Immunoreactive bands were detected by chemiluminescence (GE Healthcare). Protein levels were quantified by densitometry using ImageJ (NIH).

**Figure 6:**
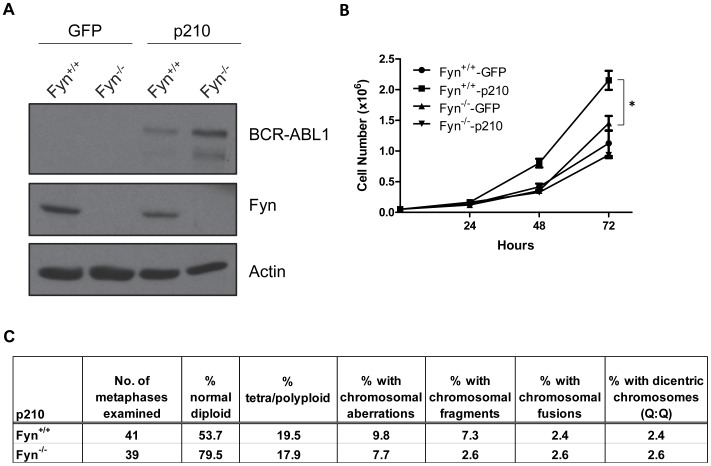
Loss of Fyn decreases the growth of MEFs transduced with BCR-ABL1 and protects against BCR-ABL1 induced genomic instability. (A) Immortalized mouse embryonic fibroblasts (MEFs) derived from Fyn wild-type (Fyn^+/+^) and Fyn knockout (Fyn^−/−^) mice were transduced with vector (GFP) or vector expressing p210 BCR-ABL1 (p210). After sorting GFP-positive cells, BCR-ABL1 and Fyn protein expression were monitored in total cell lysate by Western blot using antibodies specific for Abl and Fyn, respectively. Actin was used as a loading control. The blot is representative of three independent experiments. (B) Vector (GFP) and p210 transduced MEFs were plated in 6-well plates and live cells were counted on the days indicated. *p<0.01, n = 3, mean+SEM. (C) Table depicting the various chromosomal abnormalities seen in Fyn^+/+^ and Fyn^−/−^ MEFs transduced with BCR-ABL1.

### Immunocomplex Kinase Assay

Kinase assays were performed based on previously described protocols, with modifications [21], [26], [27]. Fyn was immunoprecipitated from 1 mg of lysate using 5 µl monoclonal Fyn antibody (Alexis, San Diego, CA) on a rotator overnight at 4°C. Protein A beads (30 µl) were added and incubated at 4°C for 4 hours. Samples were centrifuged for one minute at 6,000 rpm and 4°C. Supernatant was removed and the beads were washed twice with 500 µl lysis buffer. A final wash with 500 µl kinase buffer (25 mM Tris-HCl pH 7.4, 10 mM MgCl_2_, 1 mM DTT) was performed followed by a 20 minute kinase reaction at 30°C in 20 µl kinase cocktail (1 µCi ATP γ-32P (MP Biomedicals, Solon, OH), 2 µg Sam 68 (Santa Cruz Biotechnology, Santa Cruz, CA), and 5 µM ATP in kinase buffer. The reaction was quenched with 2X Laemmli loading buffer and samples were boiled for 5 minutes and loaded onto an 8% SDS-polyacrylamide gel. Following electrophoresis, gels were fixed for one hour in 45% methanol (v/v), 5% glacial acetic acid (v/v) solution. Gels were dried and phosphorylated protein detected by exposing the gels to film. Densitometry was performed using ImageJ (NIH).

### Measurement of DNA Synthesis

DNA synthesis was measured using an APC BrdU Flow Kit containing BrdU, an APC-conjugated BrdU antibody, 7-AAD, and washing and fixing buffers (BD Pharmingen, San Jose, CA). One million cells were pulsed for 30 minutes with 10 µM BrdU in RPMI medium to stain newly synthesized DNA strands and staining was carried out per manufacturer’s instructions.

### Benzidine Staining

The benzidine staining was performed as previously described [28]. Briefly, the benzidine stock solution contained 0.2% w/v benzidine hydrochloride in acetic acid. Prior to staining of the cells, 20 µL of hydrogen peroxide was added for each 1 mL benzidine solution (0.6% final concentration). Cells (2×10^5^) were washed once with PBS, resuspended in a 1∶1 (v/v) ratio of PBS and benzidine stain, and incubated for 2 minutes at room temperature. Benzidine positive cells were counted by light microscopy. At least 200 cells were counted for each cell line.

### RNA Analysis

Total RNA from K562 cells transduced with vector, FynWT, and constitutively active Fyn (FynY528F) was purified using RNeasy mini kit (Qiagen). Total RNA (250 ng) was reverse transcribed using the OmniScript RT Kit (Qiagen) following the manufacturer’s directions. Real-time PCR was performed on the BioRad iCycler using the iTaq Universal SYBR Green Supermix (BioRad) in a 20 µL volume containing 1 µL cDNA (diluted 1∶5), 10 µL 2x SYBR Green PCR master mix, and 1 µL of primer mix (10 µM forward primer and 10 µM reverse primer). The PCR primer sequences for human integrin β_3_ (ITGβ_3_) were previous described [29] and are as follows: ITGβ_3_: forward, 5′-CCGTGACGAGATTGAGTCA-3′ and reverse, 5′-AGGATGGACTTTCCACTAGAA-3′; β-actin: forward, 5′-CTGTGGCATCCACGAAACTA-3' and reverse, 5′-CGCTCAGGAGGAGCAATG-3'. Annealing temperatures for ITGβ_3_ and β-actin were 60°C and 58°C, respectively. The obtained Ct value for ITGβ3 was normalized to the Ct value of β-Actin. All samples were then compared to that of the untreated K562 cells.

### Cell Size and Cell Cycle Analysis

Cell size was measured by forward and side light scatter dot plots by flow cytometry. For cell cycle analysis, cells were centrifuged at 1,000 rpm for 5 min, washed with PBS, and resuspended in 500 µl of propidium iodide (PI) solution (50 µg/ml PI, 0.1%Triton-X-100, and 0.1% sodium citrate in PBS). Samples were incubated overnight at 4°C followed by flow cytometric analysis on the FL-3 channel of a Becton Dickinson FACSCalibur. CellQuest software (Becton Dickinson, Franklin Lakes, NJ) was used for cell cycle analysis. DNA fragmentation is found in the subdiploid cell populations and is indicative of apoptosis.

### Genomic Instability

Chromosomal aberrations were visualized using Giemsa staining. Seven million cells from each of the K562 cell lines were centrifuged at 1,500 rpm for 8 min and resuspended in 0.075 M KCl to induce cell swelling. Samples were incubated at room temperature for 15 minutes followed by addition of 1 ml of 3∶1 methanol:acetic acid solution. Samples were centrifuged again at 1,500 rpm for 8 minutes followed by fixing in cold 3∶1 methanol:acetic acid solution. Fixed cells were stored at −20°C until slide preparation. Cells were dropped onto slides from a height of at least six inches. Slides were allowed to dry at room temperature for approximately 3 hours. The slides were then stained with 1% Giemsa solution in water for 10 minutes and rinsed with water. Metaphase spreads were observed under a light microscope. At least 1000 nuclei per sample were scored. Genomic instability was determined by enumerating chromosomal aberrations in at least 30 metaphases per sample under a magnification of 100X.

### Statistical Analysis

Unless otherwise indicated, values in tables and figures are expressed as the mean ± SEM of at least triplicate determinations. Statistical comparisons were made using GraphPad Prism 4.0 software (GraphPad Software, Inc., La Jolla, CA) by Student’s t test. A probability value of <0.05 was considered to be significant.

## Results

### Overexpression of Constitutively Active Fyn Influences K562 Cell Proliferation

Our previously published data demonstrate that knockdown of Fyn in K562 cells causes decreased cell growth [21] which suggests that Fyn plays a role in cell growth in the context of BCR-ABL1 expression. Additionally, we observed increased Fyn phosphorylation in cells expressing BCR-ABL1 [21] which often correlates with kinase activity. However, the functional consequences of higher Fyn activity are poorly understood. Therefore, we sought to determine how overexpression of Fyn and constitutively active Fyn affected the growth and clonogenicity of K562 cells. We transduced K562 cells with MigR1 vector alone, vector expressing wild-type Fyn (Fyn WT), or vector expressing a point mutant of Fyn which leads to constitutive activation (Fyn Y528F). The Y528F mutation has been shown to stimulate the catalytic function of Fyn and activate downstream signaling pathways [30], [31], [32]. Western blot analysis on K562 cells transduced with vector, Fyn WT or Fyn Y528F demonstrates that both wild-type and constitutively active Fyn are overexpressed by almost two-fold in K562 cells (Figure 1A). We also transduced K562 with the dominant-negative form of Fyn (K295M) but were unable to detect decreases in Fyn activity in the kinase assay due to the basal activity of Fyn in the K562 cells (data not shown). To evaluate Fyn kinase activity in each cell line, immunocomplex kinase assays using Sam68 as a Fyn substrate were performed. Cells expressing wild-type Fyn display a 1.8-fold increase in kinase activity when compared to parental K562 (Figure 1B). This is consistent with degree of overexpression and suggests that some extent of auto-activation of Fyn kinase occurs. Vector transduced cells also display increase Fyn kinase activity despite no change in Fyn protein expression which may be attributed to the activation of Fyn by viruses [33], [34]. However, constitutively active Fyn transduced cells, while overexpressed to similar levels at wild-type Fyn, have an even greater increase (3.7-fold) in kinase activity relative to the parental cells (Figure 1B).

To evaluate the effects of overexpressing Fyn or increasing kinase activity on the growth of K562 cells, vector, Fyn WT, and Fyn Y528F were plated and the number of viable cells was counted after 96 hours. Interestingly, cells overexpressing wild-type Fyn showed slowed growth as compared to parental or vector transduced cells (Figure 1C). Moreover, K562 cells expressing constitutively active Fyn had significantly (p≤0.05) fewer cells by 96 hours (Figure 1C). We also measured clonogenic growth in a soft agar assay to determine how Fyn overexpression affects clonogenecity. After one week, K562 cells transduced with vector only or cells overexpressing wild-type Fyn had increased colony numbers in a soft agar colony assay as compared to the parental K562 cells (Figure 1D). This result correlates with amount of Fyn kinase activity (Figure 1B) in these lines and supports our previous observations that Fyn contributes to K562 cell growth (Figure 1C and [21]). However, consistent with the cell proliferation data, cells overexpressing Fyn Y528F have significantly (p≤0.001) fewer colonies than vector transduced cells (Figure 1D) suggesting that too much Fyn kinase activity has negative effects on cell growth.

To further evaluate how Fyn expression and activity alters proliferation, we measured DNA synthesis using BrdU incorporation assays. The expression of wild-type Fyn had little effect on the rate of proliferation however, constitutively active Fyn greatly reduced the percent of BrdU+ cells; 52.9% for Fyn WT versus 37.8% for Fyn Y528F (Figure 1E). These results are consistent with cell growth and colony formation assays and suggest that high levels of Fyn kinase activity have negative effects on cell proliferation and growth.

### Constitutive Activation of Fyn Alters Cell Size but does not Influence Erythroid or Megakaryocytic Differentiation

In addition to effects on cell proliferation, we observed that cells expressing constitutively active Fyn were noticeably larger than vector or Fyn WT cells. We further evaluated differences in cell size by measuring the mean forward scatter by flow cytometry and diameter by Vi-Cell of the live cell population. As depicted in the dot plot in Figure 2A, Fyn Y528F cells showed increased mean forward scatter, indicated by the shift in the total live cell population to the right, as compared to vector transduced K562 cells. Cells expressing constitutively active Fyn (Fyn Y528F) had an average mean forward scatter value of 640 whereas vector cells averaged 520 (Figure 2D, p≤0.05). Overexpression of wild-type Fyn also showed a trend towards increased cell size when compared to vector (Figure 2B). We also used a Vi-CELL to measure the average cell diameter and obtained similar results (data not shown). These data indicate that Fyn activity controls pathways that regulate cell size.

One factor that contributes to cancer cell proliferation and changes in cellular size is the induction of differentiation. To evaluate whether the increased cell size observed by overexpression of constitutively active Fyn was due to differentiation, we measured erythroid and megakaryocytic differentiation by benzidine staining or monitoring expression of integrin β_3_ (ITGβ_3_), a previously described megakaryocyte-specific cell surface marker [35], [36], respectively. Expression of constitutively active Fyn did not affect the basal number of erythroid cells (benzidine positive cells) or megakaryocytes (ITGβ_3_ positive cells) (data not shown). Previous studies have demonstrated that K562 cells can be used as a model system to study factors that regulate leukemic cell differentiation and can be treated with hemin or phorbol myristate acetate (PMA) to induce erythroid or megakaryocytic differentiation, respectively [37], [38]. Treatment of K562 cells overexpressing Fyn or constitutively active Fyn with 40 µM hemin (Figure 2C) or 10 nM PMA (Figure 2D) for three days showed no difference in the induction of markers of erythroid or megakaryocytic differentiation. These data suggest that the increase in cell size is not due to increased differentiation of the cells.

### Elevated Fyn Kinase Activity in CML Cells Causes Additional Increases in Genomic Instability

As DNA content increases and cell cycle progresses, cells increase in size. The decreased growth and clonogenicity, and increase in cell size, of constitutively active Fyn expressing cells may be caused by the role of Fyn in regulating the cell cycle. In fact, Fyn has been implicated as a mediator of mitosis and meiosis [39], [40], [41]. To determine whether expression of constitutively active Fyn caused cell cycle alterations, we evaluated the cell cycle profiles of the Fyn WT and constitutively active expressing K562 cells by propidium iodide staining and analysis by flow cytometry. Quantification of the percent of cells in G1, S, and G2/M phases of the cell cycle revealed no statistically significant differences between vector, Fyn WT, and Fyn Y528F expressing K562 cells (data not shown). However, Fyn Y528F expressing cells displayed an increase in the percent of cells with >4N DNA content (9.33±1.13%) as compared to Fyn WT or vector controls (1.91±0.18 and 5.12±0.47%, respectively; Figure 3A and B). These results indicate that Fyn Y528F cells, which are larger in size, also harbor increased DNA content. This suggests that while constitutively active Fyn does not influence the cell cycle it does contribute to the accumulation of DNA content.

The increased number of cells expressing constitutively active Fyn with >4N DNA content indicates that Fyn activity may influence genomic stability. It has been suggested that certain stresses can lead to cell death in susceptible cells but contribute to genomic instability in surviving cells which, following selective pressure, leads to enhanced progression of disease [42]. To further investigate the effects of Fyn overexpression on the accumulation of genomic alterations in K562 cells, we stained the surviving population of cells with Giemsa and quantified the number of diploid and polyploidy cells as well as various other chromosomal aberrations, including chromatid breaks, chromosome fragments, chromosome fusions, dicentric chromosomes, and marker chromosomes. A representative image of Fyn Y528F expressing cells is shown in Figure 3C and depicts several chromosomal breaks. Analysis of at least 35 metaphases per sample revealed that cells overexpressing both Fyn WT and Fyn Y528F show an increased percentage of polyploidy cells, 35.7 and 42.1%, respectively (Figure 3D). Interestingly, Fyn Y528F expressing cells had a higher percentage of chromosomal aberrations in comparison to the others (15.1%) and include chromosomal fragments (1.4%), chromosomal fusions (8.2%), and dicentric chromosomes (6.9%) (Figure 3D). These data are striking and suggest that elevated Fyn expression may contribute to the increased genomic instability observed in blast crisis CML.

### Genetic Loss of Fyn Decreases the Proliferation and Clonogenic Potential of BCR-ABL1 Expressing Cells

Our above studies suggest that increased Fyn activity may contribute the genomic instability observed in blast crisis CML. Additionally, our previous studies demonstrated that Fyn is upregulated in BCR-ABL1 expressing CML cells and shRNA knockdown of Fyn leads to decreased growth of these cells [21]. To further examine the role of Fyn in BCR-ABL1-mediated proliferation, clonogenicity, and genomic instability, we transduced bone marrow cells derived from wild-type or Fyn knockout mice with the MigR1 retroviral vector expressing either GFP or the p210 BCR-ABL1 oncogene. GFP+ cells were sorted by FACS and Western blot analysis was performed to confirm the expression of Fyn and p210 in these cells (Figure 4A). We then monitored the ability of these cells to form colonies in a soft agar assay. As expected, p210 transduced cells have significantly increased colony numbers in both cell lines compared to their vector transduced counterparts (Figure 4B). Importantly, while genetic loss of Fyn has little to no effect on vector transduced cells, there is a 40% decrease in the number of colonies formed by Fyn-deficient p210 expressing cells as compared to wild-type p210 expressing cells (Figure 4B). Additionally, loss of Fyn results in decreased cell growth of bone marrow cells expressing p210 (Figure 4C).

### Overexpression of Constitutively Active Fyn in Fyn-deficient Bone Marrow Cells Negatively Impacts Growth and Proliferation

K562 cells are known to contain elevated levels of Fyn protein due to the expression of BCR-ABL1 [21]. To determine whether the effects of Fyn kinase activity on cell growth and proliferation occurred only in the context of BCR-ABL1, we transduced bone marrow cells from Fyn-deficient mice with retrovirus containing vector, Fyn WT, or Fyn Y528F. Both wild-type Fyn and constitutively active Fyn are expressed at similar levels in these cells (Figure 5A). We then tested the clonogenic properties of these cells in a soft agar assay. Interestingly, as observed in BCR-ABL1 expressing K562 cells, overexpression of Fyn Y528F leads to significantly fewer colonies formed (p≤0.001) in comparison to vector or Fyn WT expressing cells (Figure 5B). Collectively, these data suggest that constitutive activation of Fyn acts as a stress that reduces cellular proliferation and clonogenic potential.

### Genetic Loss of Fyn Reduces Genomic Instability Induced by BCR-ABL1

To further demonstrate a role for Fyn in regulating cellular proliferation, we utilized immortalized mouse embryonic fibroblast (MEF) cells derived from wild-type and Fyn-knockout mice transduced with vector expressing GFP or p210. Similar to our results in bone marrow cells, Fyn-deficient MEFs transduced with p210 display reduced growth as compared to wild-type p210 expressing MEFs (Figure 6B) despite their elevated expression of BCR-ABL1 (Figure 6A). Interestingly, in contrast to our previous studies [21], Fyn expression in wild-type MEFs transduced with BCR-ABL1 remained unchanged (Figure 6A). This is likely due to the lack of induction of mediators, such as reactive oxygen species, Egr-1, and p47phox, which we previously identified as being important for the upregulation of Fyn [22] (data not shown). These results demonstrate BCR-ABL1 transduced MEFs may provide a model system for analyzing the roles of Fyn in cell growth and development that are independent of the induction of ROS.

To further support a role for Fyn in the maintenance of genomic integrity, we analyzed the MEF cells transduced with BCR-ABL1 for markers of genomic instability. While some markers of genomic instability remain unchanged (% tetra−/polyploidy, chromosomal fusions, and dicentric chromosomes (Q:Q)), loss of Fyn reduces the number of cells with chromosomal aberrations and chromosomal fragments (Figure 6C). Importantly, Fyn wild-type and deficient MEFs showed no difference in the amount of baseline genomic instability by the same measures used in Figure 6C (data not shown). These data indicate that genomic instability induced by BCR-ABL1 is dependent, in part, on Fyn expression. Since genomic instability has been associated with blast crisis CML [6], [7], these results warrant further study of Fyn kinase activity in CML disease progression.

## Discussion

Src family kinases have been implicated in CML [9], [10] and we have previously shown that Fyn expression increases specifically in blast crisis CML. Additionally, knockdown of Fyn leads to decreased cell growth and proliferation in vitro and in vivo [21]. Consistent with our previous studies, complete loss of Fyn using genetic knockout models decreases the proliferation and clonogenic potential of cells transduced with BCR-ABL1 (Figures 1 and 4) underscoring a dependency upon Fyn for BCR-ABL1 mediated growth and clonogenicity. Here, we report the effects of Fyn kinase on the growth and proliferation of BCR-ABL1 expressing cells. Fyn has been shown to associate with BCR-ABL1 and regulate its signaling through phosphorylation of the SH2 and SH3 domains suggesting that Fyn kinase activity is elevated in CML [21], [43]. However, the cellular effects of Fyn kinase are not well described.

Using a cell line model of blast crisis CML, we discovered that overexpression of constitutively active Fyn caused increased aneuploidy and genomic alterations (Figure 3). We also observe fewer markers of genomic instability in BCR-ABL1 expressing cells which lack Fyn expression (Figure 6). These data suggest a novel role for Fyn kinase activity in regulating genomic instability, a common feature of blast crisis CML. Interestingly, while similar levels of Fyn expression are achieved (Figures 1A and 5A), elevated Fyn kinase activity had deleterious effects on the growth of K562 cells (Figure 1C–E) and bone marrow cells derived from Fyn knockout mice (Figure 5B) instead of further enhancing mitogenic signals and promoting cell growth and survival. One possible explanation for these observations is that constitutive Fyn kinase activity leads to damage that, over time, causes mutations that kill susceptible cells but allows for mutations that are selected for during the progression of disease. In addition, these results suggest that a balance of Fyn expression and activity exist to regulate cell growth. Similar thresholds of expression and activity are seen for other oncoproteins, such as Ras. Several studies have demonstrated that forced expression of H-Ras induces cellular senescence even though activated Ras can cause malignant transformation [44], [45], [46], [47], [48], [49], [50].

Several lines of evidence suggest the importance of Fyn in the regulation of cell cycle [17], [51], [52]. Our data demonstrate that the overexpression of constitutively active Fyn leads to increased cell size and genomic instability (Figure 2 and 3). Moreover, loss of Fyn reduced the number of genomic abnormalities induced by BCR-ABL1 (Figure 6). Genomic alterations can occur from cell cycle irregularities, such as mitotic arrest or malfunctioning checkpoints. Cells with checkpoint defects are able to survive with a higher frequency of aneuploidy [53]. Mitotic abnormalities in aneuploid cancer cells have been frequently observed, including abnormalities in kinetochore components, spindle checkpoint proteins, and centrosomes [54]. Centrosome abnormalities, including multiple centrosomes per cell, have been observed in CML patients prior to the occurrence of secondary chromosomal aberrations [55]. Moreover, it has been shown that Src and Fyn are both required in the G_2_ phase for fibroblasts to undergo cell division [17]. Fyn has also been found to localize to meiotic and mitotic spindles in rat oocytes [56] and to the mitotic spindle in T cells [57], yet its precise function at these sites is unknown. Further studies need to be conducted to uncover substrates of Fyn that contribute to regulation of cell cycle and genomic instability in CML.

The data presented here extend our previous findings that Fyn mRNA and protein expression is increased in BCR-ABL1 expressing cell lines, mouse models, and patient samples [21]. A recent report examining Fyn mRNA expression in CML patient samples contradicts our observations however, the authors did not evaluate Fyn protein expression and used different viral constructs for BCR-ABL1 overexpression [58]. This could be the reason for the discrepancy since it has been reported that certain viruses influence Fyn [33], [34] yet, the authors did not evaluate the effects of viral transduction on the parental cells to ensure that Fyn expression was unchanged. Supporting our observation are several independent reports demonstrating a role for Fyn in CML progression and imatinib resistance. Microarray analysis of imatinib resistant cells revealed upregulation of Fyn gene expression and knockdown of Fyn in these cells resensitized them to imatinib [23]. Moreover, activation of the Fyn/ERK signaling axis was also shown to mediate imatinib resistance [59]. In our current study, we demonstrate a role for Fyn kinase activity in the accumulation of genomic instability that is associated with blast crisis by investigating the effects of constitutive activation of Fyn in K562 cells. Taken together, these data implicate Fyn in the progression of CML and provide the rationale for our current studies evaluating the correlation between Fyn expression and activity in CML patient specimens.

Second generation tyrosine kinase inhibitors, such as dasatinib, have been developed to more potently inhibit BCR-ABL1 and overcome resistance developed toward imatinib. In Phase 3 studies evaluating the efficacy of dasatinib in patients with newly diagnosed blast crisis CML, dasatinib showed significantly higher levels of complete cytogenetic response and molecular responses [60]. The increased efficacy of dasatinib in these patients could be due to the ability of dasatinib to effectively block Src family kinase activity, including Fyn [61]. In addition, our data suggests that Fyn expression, as well as activity, may be an attractive target for CML therapies. We have shown that early growth response 1 (Egr-1) contributes to Fyn upregulation in CML cells [22]. Therefore, targeting Egr-1 may be one strategy for reducing Fyn expression. Another strategy involves understanding the kinase-independent roles of Fyn in promoting CML disease progression. Protein-protein interactions, some of which are facilitated by the SH2 and SH3 domains of Fyn, may promote binding and activation of other molecules important in BCR-ABL1 signaling. For example, it has been demonstrated that Fyn associates with γ-tubulin, a protein found within centrosomes [62]. Sam68 is another protein that associates with Fyn and is important in regulating cell cycle transitions, including mitosis [63]. In addition, the SH2 and SH3 domains of the Src family kinases are essential for repression of enzyme activity and deletion of these domains leads to the constitutive activation and oncogenic potential [64], [65], [66], [67]. Further studies are necessary to determine the relevance of Fyn substrate phosphorylation versus its function as an adaptor molecule in the progression to blast crisis CML. Such studies will reveal novel molecular targets for the treatment of patients in blast crisis or for preventing progression from chronic/accelerated phase to blast crisis.
